# Progress Update and Challenges on $\dot{\text{V}}$O_2max_ Testing and Interpretation

**DOI:** 10.3389/fphys.2020.01070

**Published:** 2020-09-03

**Authors:** Marcos Martin-Rincon, Jose A. L. Calbet

**Affiliations:** ^1^Department of Physical Education, University of Las Palmas de Gran Canaria, Las Palmas de Gran Canaria, Spain; ^2^Research Institute of Biomedical and Health Sciences (IUIBS), University of Las Palmas de Gran Canaria, Las Palmas de Gran Canaria, Spain; ^3^Department of Physical Performance, The Norwegian School of Sport Sciences, Oslo, Norway

**Keywords:** calibration, cardiopulmonary exercise testing, ramp exercise, indirect calorimetry, reproducibility, breath-by-breath, metabolic cart, performance

## Abstract

The maximal oxygen uptake ($\dot{\text{V}}$O_2max_) is the primary determinant of endurance performance in heterogeneous populations and has predictive value for clinical outcomes and all-cause mortality. Accurate and precise measurement of $\dot{\text{V}}$O_2max_ requires the adherence to quality control procedures, including combustion testing and the use of standardized incremental exercise protocols with a verification phase preceded by an adequate familiarization. The data averaging strategy employed to calculate the $\dot{\text{V}}$O_2max_ from the breath-by-breath data can change the $\dot{\text{V}}$O_2max_ value by 4–10%. The lower the number of breaths or smaller the number of seconds included in the averaging block, the higher the calculated $\dot{\text{V}}$O_2max_ value with this effect being more prominent in untrained subjects. Smaller averaging strategies in number of breaths or seconds (less than 30 breaths or seconds) facilitate the identification of the plateau phenomenon without reducing the reliability of the measurements. When employing metabolic carts, averaging intervals including 15–20 breaths or seconds are preferable as a compromise between capturing the true $\dot{\text{V}}$O_2max_ and identifying the plateau. In training studies, clinical interventions and meta-analysis, reporting of $\dot{\text{V}}$O_2max_ in absolute values and inclusion of protocols and the averaging strategies arise as imperative to permit adequate comparisons. Newly developed correction equations can be used to normalize $\dot{\text{V}}$O_2max_ to similar averaging strategies. A lack of improvement of $\dot{\text{V}}$O_2max_ with training does not mean that the training program has elicited no adaptations, since peak cardiac output and mitochondrial oxidative capacity may be increased without changes in $\dot{\text{V}}$O_2max_.

## Introduction

### Relevance of $\dot{\text{V}}$O_2max_ Determination in Performance and Health

The maximal oxygen uptake ($\dot{\text{V}}$O_2max_) is the highest flow of oxygen (O_2_) that can be used by the organism, representing the integrated capacity of the pulmonary, cardiovascular and muscle systems to take up, transport and utilize O_2._ Classically, the determination of the $\dot{\text{V}}$O_2max_ has been considered the gold standard for the assessment of the functional limits of the cardiorespiratory system (Taylor et al., 1955; Mitchell et al., 1958; Di Prampero, 2003; Levine, 2008). The $\dot{\text{V}}$O_2max_ is one of the main factors associated with endurance performance (Joyner and Coyle, 2008) in various exercise modalities (Magel and Faulkner, 1967; Holmér et al., 1974; Maughan and Leiper, 1983). When a wide range of performances is included, $\dot{\text{V}}$O_2max_ is the single best predictor of performance as shown in trained non-elite athletes, in whom $\dot{\text{V}}$O_2max_ explained 90.2% of the total variance in a 16 km running time trial (McLaughlin et al., 2010). Although $\dot{\text{V}}$O_2max_ does not predict performance in homogeneous groups of athletes (i.e., elite level), an exceptionally high $\dot{\text{V}}$O_2max_ constitutes a prerequisite to compete at world-class level (Joyner and Coyle, 2008; Losnegard et al., 2013).

The terms cardiorespiratory fitness and $\dot{\text{V}}$O_2max_ are used interchangeably, particularly in epidemiological studies. The assessment of $\dot{\text{V}}$O_2max_ is gaining interest in clinical populations since it constitutes the strongest predictor of all-cause mortality when compared with other risk factors such as hypertension, smoking, obesity and diabetes (Myers et al., 2002; Lee et al., 2010). Furthermore, $\dot{\text{V}}$O_2max_ is one of the objective criteria used to select candidates for heart transplantation (Mehra et al., 2016) and predicts the success of thoracic surgery (Brunelli et al., 2014). Thus, both in sport science and clinical medicine fields, there is a necessity for an accurate and precise determination of $\dot{\text{V}}$O_2max_.

## Assessment of $\dot{\text{V}}$O_2max_: Evolution of Procedures and the Plateau Phenomenon

Hill and Lupton (1923) are credited for performing the first assessments of $\dot{\text{V}}$O_2max_. Years later, Taylor et al. (1955) proposed standardized procedures to determine $\dot{\text{V}}$O_2max_, which were based on discontinuous protocols using three-min constant-intensity exercise periods carried out on subsequent days. At that time, $\dot{\text{V}}$O_2_ measurements were based on the use of Douglas Bags and Tissot spirometers (Macfarlane, 2001) combined with Haldane’s or Scholander’s methods to obtain the concentration of O_2_, which turned to be the gold-standard method for the assessment of $\dot{\text{V}}$O_2max_. Taylor et al. (1955) defined the “plateau” in the $\dot{\text{V}}$O_2_/intensity relationship as an increase in $\dot{\text{V}}$O_2_ lower than 150 mL.min^–1^ or ∼2 mL.kg^–1^.min^–1^ with increasing exercise intensity. Continuous exercise protocols which allow for the assessment of additional variables of cardiorespiratory fitness were developed during the years to follow (Balke and Ware, 1959; Bruce, 1971; Froelicher et al., 1974a,b). However, these continuous protocols often failed to fulfill Taylor’s criteria for the plateauing effect (Froelicher et al., 1974a,b; Pollock et al., 1976).

The development of fast gas analyzers and flow sensors during the 1960s and 1970s together with the progressive miniaturization of computers resulted in the proposal of shorter continuous protocols which used the “limit of tolerance” of the subject (volitional exhaustion) as the criterion for test finalization (Whipp et al., 1981; Buchfuhrer et al., 1983). The duration of continuous protocols was judged as a critical variable for the achievement of “true” maximal $\dot{\text{V}}$O_2max_ values (Buchfuhrer et al., 1983). These authors reported that durations between 8 and 17 min yielded the highest $\dot{\text{V}}$O_2max_ values in five low to moderately trained subjects during treadmill and cycling ergometry, intuitively proposing 10 ± 2 min as the ideal duration. A critical factor that determines the length of the test is the rate at which intensity is increased over time or ramp slope, usually expressed in Watts/min (or speed/min). Research carried out in the last 40 years has confirmed that a similar $\dot{\text{V}}$O_2max_ can be attained despite wide differences in the magnitude of the increments and duration of the tests (Zhang et al., 1991; Takaishi et al., 1992). Midgley et al. (2008) concluded that durations of 5 to 26 min could be optimal to achieve $\dot{\text{V}}$O_2max_ in healthy individuals, provided that those with shorter durations are preceded by proper warm-up. Moreover, even repeated all-out sprint exercise has been shown to allow for the achievement of true $\dot{\text{V}}$O_2max_ values, particularly when an active recovery is applied during the resting periods (Dorado et al., 2004; Gelabert-Rebato et al., 2018).

The utilization of gas exchange indirect calorimetry evolved rapidly from the Douglas Bag into technologies capable of examining the time course of gas exchange during exercise more comprehensively, namely mixing chambers and breath-by-breath systems. However, the Douglas Bag method is still considered as the gold-standard method (Shephard, 2017). Mixing-chamber automated systems measure expired gas fractions from several breaths collected into a small chamber (Wilmore and Costill, 1974; Wilmore et al., 1976), while breath-by-breath technology analyses the gas content of each breath by measuring instantaneous expiratory flow in conjunction with simultaneous tidal concentrations of exhaled gases (Beaver et al., 1973, 1981). Modern breath-by-breath metabolic carts are equipped with fast responding O_2_ and CO_2_ sensors and permit a precise and accurate assessment of the end-tidal O_2_ and CO_2_ pressures (Losa-Reyna et al., 2015). The latter can be used in conjunction with pulse oximetry for an indirect evaluation of pulmonary gas exchange, thus enhancing the utility of ergometry in clinical populations (Macfarlane, 2001; Sun et al., 2002).

Nevertheless, several technical limitations may undermine the precision and accuracy of breath-by-breath analyses if not adequately addressed (Di Prampero and Lafortuna, 1989). Yet, with state-of-the-art, well-calibrated and fast-responding metabolic carts, it is possible to closely match the levels of accuracy and precision of the Douglas bag method (Medbo et al., 2012; Nieman et al., 2013; Perez-Suarez et al., 2018; Martin-Rincon et al., 2019).

## Confirming the Attainment of $\dot{\text{V}}$O_2max_: From the Plateau Phenomenon to the Necessity of a Verification Phase

Despite considerable technical improvements in metabolic carts, ergometers and exercise protocols, there is yet no universal agreement on the criteria for the attainment of a “true” $\dot{\text{V}}$O_2max_. Until recent years, the most referenced criteria to confirm $\dot{\text{V}}$O_2max_ attainment has been the presence of a plateau or leveling off in $\dot{\text{V}}$O_2_, even though several investigations have shown that the incidence of the plateau is population-dependent (Achten et al., 2002; Huggett et al., 2005) and may not be manifested despite the attainment of a valid $\dot{\text{V}}$O_2max_ (Day et al., 2003; Rossiter et al., 2006). In addition to the 150 mL or 2 mL.kg^–1^.min^–1^ criterion previously proposed by Taylor et al. (1955) for the attainment of the plateau, other cut-off values have been postulated, as the Δ$\dot{\text{V}}$O_2_ < 80 mL.min^–1^ proposed by Astrand (1960) or the presence of a slope not different from zero with an increase in exercise intensity (Myers et al., 1990). However, the efficacy of these criteria to detect the plateau depends on the specific exercise protocol (Duncan et al., 1997), the population studied (Doherty et al., 2003; Beltrami et al., 2014), the subjects’ experience (Gordon et al., 2015) and the breath-by-breath averaging strategy (Astorino, 2009; Nolan et al., 2014; Martin-Rincon et al., 2019). For example, attainment of a plateau may require to exercise with a marked recruitment of anaerobic metabolism during 1–3 min, entailing a high perception of effort and a strong central command to avoid task failure (Torres-Peralta et al., 2016a,b). Not surprisingly, subjects achieving a plateau present 4–5% higher levels of pulmonary ventilation (an indication of higher central command activation), respiratory exchange ratio (RER), and blood lactate concentration than those not reaching a plateau (Edvardsen et al., 2014). Given the limited duration of the plateau phase, its identification is facilitated by using shorter (15, 30 s) than longer (i.e., 60 s) time-averaging strategies (Astorino, 2009; Nolan et al., 2014).

This is further complicated by the influence that different exercise protocols and modes (Millet et al., 2009) may have on the highest $\dot{\text{V}}$O_2_ value attainable (Midgley et al., 2008). Consequently, when uncertainty exists regarding whether a $\dot{\text{V}}$O_2max_ value is real or not, the highest $\dot{\text{V}}$O_2_ recorded is named $\dot{\text{V}}$O_2peak_. Since many individuals reach a higher $\dot{\text{V}}$O_2max_ value during a supramaximal rather than during an incremental exercise test, it is necessary to include a verification test few minutes after the end of the incremental test (Poole and Jones, 2017), even in clinical populations (Moreno-Cabanas et al., 2020).

## Recommended Procedures for Quality Control of Metabolic Carts

Quality control procedures are mandatory to avoid technical errors. Most advanced metabolic carts are equipped with automated calibration routines that should be followed according to the recommendations of the manufacturers and using high-grade calibration O_2_ and CO_2_ gases. Besides the standard automated calibration, the flow sensors should be checked regularly with calibration syringes or commercially available metabolic simulators. Proper maintenance and replacement of flowmeters, O_2_ cells, Nafion tubing and any other tubing and valves should follow the recommendations of the manufacturers. In addition, combustion tests of pure fuels such as methanol, butane or propane can be used to perform an integral check on the precision and accuracy of the metabolic carts. The combustion tests have the advantage of generating heat and some moisture, similar to the combustion present in living organisms (see, for example, Perez-Suarez et al., 2018). It is also advisable to perform regular checks at low and high exercise intensities, for example, on laboratory members holding a steady physical fitness level, as an additional biological check.

Verification of the performance of ergometers and treadmills is also necessary. In addition to the recommendations made by manufacturers, cross-validation biological checks with other ergometers are recommended. For example, repeated cardiorespiratory measurements at target intensities in the same person can be used to verify the stability of physiological variables. Treadmill speed and inclination can be easily verified manually or with odometer wheels.

## Impact of Data Processing Strategies on the $\dot{\text{V}}$O_2_ Imputed as the $\dot{\text{V}}$O_2max_

**FIGURE 1:**
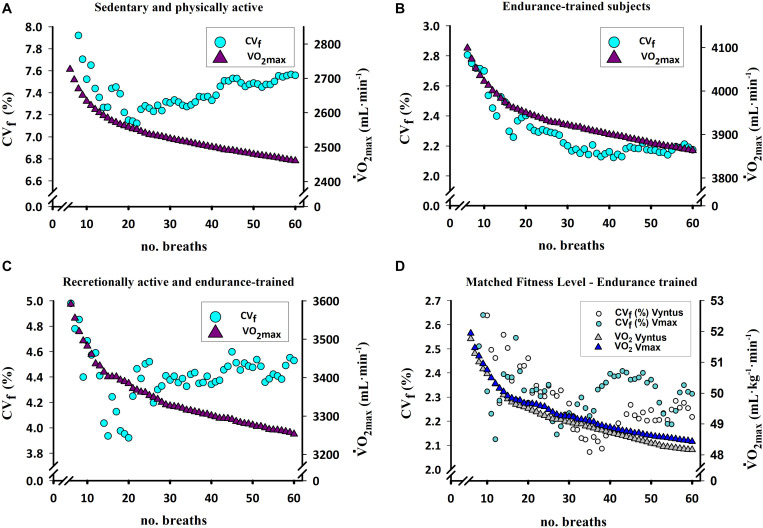
Change in the $\dot{\text{V}}$O_2max_ value and its reproducibility in subjects divergent for fitness status using two different automated metabolic carts operated in breath-by-breath mode with different rolling breath-averages. **(A)** $\dot{\text{V}}$O_2max_ response in a heterogeneous group of sedentary and physically active overweight and obese subjects ($\dot{\text{V}}$O_2max_ range: 13–40 mL/kg/min) assessed with Vmax N29 Sensormedics (*n* = 51). **(B)** $\dot{\text{V}}$O_2max_ response in a group of endurance-trained subjects ($\dot{\text{V}}$O_2max_ range: 40–60 mL/kg/min) assessed with Vyntus CPX (*n* = 11). **(C)** $\dot{\text{V}}$O_2max_ response in a group of recreationally active and endurance-trained subjects ($\dot{\text{V}}$O_2max_ range: 35–60 mL/kg/min) performing one test with Vmax N29 and a duplicate test with Vyntus CPX in random order (*n* = 11). **(D)** $\dot{\text{V}}$O_2max_ response assessed with the Vmax N29 and Vyntus metabolic carts in two groups of nine subjects each of similar $\dot{\text{V}}$O_2max_ ($\dot{\text{V}}$O_2max_ range: 40–60 mL/kg/min) **(B)**. CV_f_ (%), coefficient of variation calculated as proposed by Forkman (2009). Note the clear trend for lower reproducibility and a larger decay of the $\dot{\text{V}}$O_2max_ the lower the fitness of the subjects. Modified from Martin-Rincon et al. (2019) with kind permission.

**FIGURE 2:**
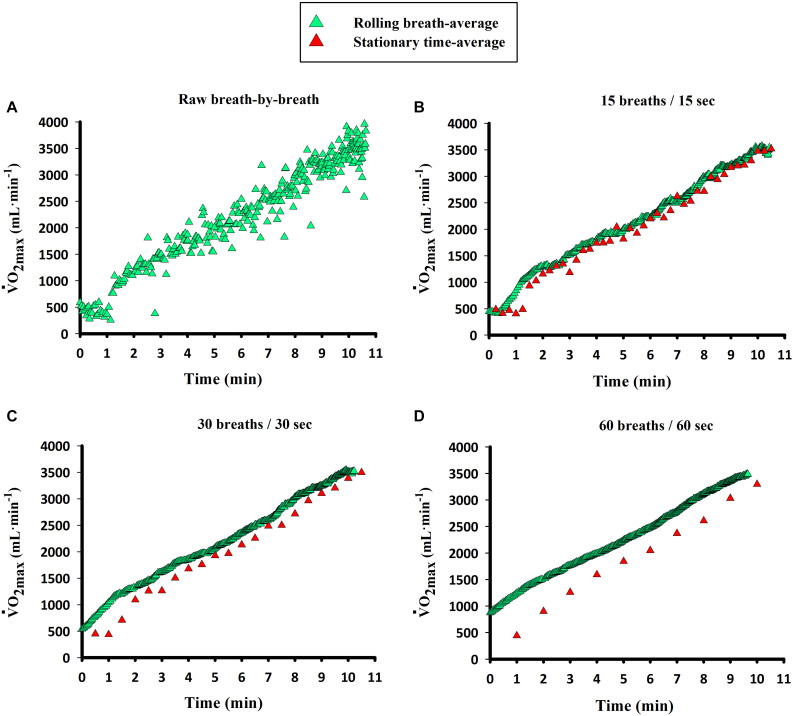
Representative $\dot{\text{V}}$O_2_ data during an incremental exercise to exhaustion in one demonstrative subject using different averaging blocks (breaths and seconds). Calculation of averaging blocks for the breath-based strategy refers to a “rolling” breath average, while time-based data were stationary time-averaged, both with the corresponding length of the block (e.g., 10 breaths or 10 s). Data are presented as **(A)** raw breath-by-breath, **(B)** 15 breaths and 15 s, **(C)** 30 breaths and 30 s, **(D)** 60 breaths and 60 s. Note the slightly higher values of breath-based averages compared to time-based averages of the same number, although a high concordance (concordance correlation coefficient = CCC > 0.97) is present. Modified from Martin-Rincon et al. (2019) with kind permission.

Until recently, not much attention had been paid to the influence of the averaging interval or strategy on the $\dot{\text{V}}$O_2_ value imputed as $\dot{\text{V}}$O_2max_ and its reproducibility (Myers et al., 1990; Hill et al., 2003; Midgley et al., 2007; Astorino, 2009; Nolan et al., 2014; Smart et al., 2015; Dideriksen and Mikkelsen, 2017). Despite the inherent high variability between breaths, there is no universal consensus on how to average indirect calorimetry data (Robergs et al., 2010). We have recently demonstrated that the $\dot{\text{V}}$O_2_ value imputed as $\dot{\text{V}}$O_2max_ can fluctuate between 4 and 10% depending on the averaging strategy and the fitness levels (Martin-Rincon et al., 2019; Figure 1). We have shown that in subjects with a $\dot{\text{V}}$O_2max_ lower than ∼40 mL/kg/min (13–40 mL/kg/min), the $\dot{\text{V}}$O_2max_ value is ∼10% higher when using shorter (i.e., 10 breaths or seconds) averaging strategies rather than longer (i.e., 60 breaths or seconds in averaging block). This effect was slightly smaller (∼6.5%) in higher fitness ($\dot{\text{V}}$O_2max_ between 40 and 60 mL/kg/min) subjects. Similarly, from a short (i.e., 10 breaths or seconds) to an intermediate length in the averaging block (i.e., 30 breaths or seconds), the decrease is ∼7.5% in low-fit and ∼5% in those with higher fitness. Therefore, with shorter averaging strategies the $\dot{\text{V}}$O_2_ value considered as $\dot{\text{V}}$O_2max_ is larger, with this effect being more marked in untrained subjects, regardless of the use of breath- or time-based averaging strategies (Martin-Rincon et al., 2019). The time- and breath-averaged $\dot{\text{V}}$O_2_ values produce similar results for a given length of the averaging block in breaths or seconds (Martin-Rincon et al., 2019; Figure 2). The values of $\dot{\text{V}}$O_2max_ obtained with different averaging strategies can be interconverted with specific equations (Martin-Rincon et al., 2019). Given the fact that the $\dot{\text{V}}$O_2max_ can be maintained only for a short time, the identification of the plateau is facilitated by short averaging intervals, as previously mentioned, without a negative impact of shorter averaging strategies on the reliability of the measurements (Martin-Rincon et al., 2019).

## Physiological Interpretation of the $\dot{\text{V}}$O_2max_

The $\dot{\text{V}}$O_2max_ value obtained in an exercise test should be checked against normative data (Edvardsen et al., 2013; Loe et al., 2013; Kaminsky et al., 2017), taking into consideration the exercise intensity achieved at the end of the test, exercise mode, age, sex, health status, fitness level, familiarization and the averaging strategy used to obtain the reference values. Since there is a close linear association between exercise intensity and $\dot{\text{V}}$O_2_, there are several linear equations that can be used to predict the $\dot{\text{V}}$O_2_ associated with a submaximal load as well as the $\dot{\text{V}}$O_2max_ (Storer et al., 1990; Hall et al., 2004). The predicted or normative value can be confronted with the measured one, having in mind that departures up to 20% are possible (Malek et al., 2004). In the case of large differences between measured and estimated or reference values, assessment errors should be ruled out.

Since the $\dot{\text{V}}$O_2max_ is a lumped parameter reflecting the flow of O_2_ from the atmosphere to the mitochondria, the flow in downwards steps of the transport chain can never be higher than in the precedent step. $\dot{\text{V}}$O_2max_ cannot be higher than O_2_ delivery, and O_2_ delivery cannot be higher than the O_2_ flow in the lungs. For the correct physiological interpretation of the $\dot{\text{V}}$O_2max_, it is critical to identify which the limiting step/factor is, holding in mind that the limiting factor may change with training (Zinner et al., 2016). In other words, it is essential to find out whether the limitation is mostly due to pulmonary ventilation, pulmonary gas exchange, cardiac output, muscle blood flow, arterial O_2_ content, muscle O_2_ diffusion capacity or the mitochondrial capacity to utilize O_2_. In general, in healthy people, including elite athletes, the main limiting factor for $\dot{\text{V}}$O_2max_ is O_2_ delivery (Saltin and Calbet, 2006). Nevertheless, couch potatoes may be limited by their capacity to use O_2_, which can be readily tested by performing an incremental exercise test in hyperoxia (Cardus et al., 1998). Optimally, during the exercise test, the arterial hemoglobin saturation should be assessed indirectly by pulse oximetry. It is important to account for the impact that blood hemoglobin concentration may have on $\dot{\text{V}}$O_2max_, and thus it is convenient to measure this variable before the tests (Calbet et al., 2002).

To facilitate the interpretation of results, the $\dot{\text{V}}$O_2max_ should always be reported in absolute terms (i.e., mL.min^–1^), together with the protocol used for its assessment and the averaging strategy. The $\dot{\text{V}}$O_2max_ value adjusted for body weight has different meanings depending on the body composition, although the relative value (i.e., $\dot{\text{V}}$O_2max_ in mL.kg^–1^.min^–1^) predicts better performance than the absolute value in competitions lasting from 60 to 90 s to several hours (Joyner and Coyle, 2008). In training studies and clinical interventions, the absolute value should imperatively be reported such that a mechanistic explanation for the changes in $\dot{\text{V}}$O_2max_, connected to the limiting factors, can be elaborated. Lack of improvement in $\dot{\text{V}}$O_2max_ with training does not mean that the training program has failed or that it has elicited no adaptations. Actually, in elite athletes, peak cardiac output may be increased, as well as mitochondrial oxidative capacity, without changes in $\dot{\text{V}}$O_2max_, due to an impairment of pulmonary gas exchange caused by the reduction of capillary blood mean transit time in the lung (Skattebo et al., 2020a). Moreover, peripheral adaptations may enhance the $\dot{\text{V}}$O_2peak_ and O_2_ extraction during small mass exercise without increasing the $\dot{\text{V}}$O_2max_ measured during whole-body exercise (Skattebo et al., 2020b). Meta-analyses on the effects of an intervention on $\dot{\text{V}}$O_2max_ should be based on absolute values, after taking into consideration the averaging strategies used in each study and the exercise modality, as previously discussed (Martin-Rincon et al., 2019).

## Future Perspectives

Although the $\dot{\text{V}}$O_2max_ value varies depending on the averaging strategy, the fact that the values obtained with different averaging strategies are closely related allows the assumption that they hold a similar physiological meaning. However, the fact that $\dot{\text{V}}$O_2max_ increases log-linearly with the shortening of the averaging interval (breath or time) (Martin-Rincon et al., 2019) implies that the real $\dot{\text{V}}$O_2max_ value should be measured with a small number of breaths or a short time interval. Since the reproducibility is not significantly lowered by reducing the averaging interval between 6 and 60 breaths (or 6 and 60 s) (Martin-Rincon et al., 2019), the most appropriate approach would be using the shortest interval, since only one true $\dot{\text{V}}$O_2max_ value must exist. From a physiological perspective it is not the same to compute the $\dot{\text{V}}$O_2max_ value with a 6-s than a 60-s averaging strategy, since the latest may be 4–10% lower, depending on the fitness status. Given the relatively low trainability of $\dot{\text{V}}$O_2max_ (Montero and Lundby, 2017), scientists and clinicians may easily misjudge the real condition of patients or the effects elicited by an intervention without accounting for the impact of the averaging strategy. Imputing an underestimated $\dot{\text{V}}$O_2max_ has important implications for integrative physiology and pathophysiology. This is the case when comparing the $\dot{\text{V}}$O_2max_ with maximal mitochondrial respiration values obtained *in vitro*, where the values of $\dot{\text{V}}$O_2max_ used to represent the whole-body $\dot{\text{V}}$O_2max_ will be smaller, and the excess mitochondrial respiratory capacity will be overestimated. Since the $\dot{\text{V}}$O_2max_ is used to calculate cardiac output by the direct Fick method, for a given systemic arteriovenous O_2_ (a-vO_2_) difference, a $\dot{\text{V}}$O_2max_ value obtained with a longer averaging interval will result in a lower calculated cardiac output by the Fick method. Likewise, the calculated pulmonary O_2_ diffusing capacity would also be greater the higher the $\dot{\text{V}}$O_2max_ imputed. Moreover, defining the $\dot{\text{V}}$O_2max_ with a shorter averaging interval results in a value that can be maintained for a shorter time, affecting the procedures to determine the velocity or intensity at $\dot{\text{V}}$O_2max_ (Billat and Koralsztein, 1996; Billat et al., 1999).

In the case that the $\dot{\text{V}}$O_2max_ is used to calculate the systemic a-vO_2_ difference using the indirect Fick method, attributing a higher $\dot{\text{V}}$O_2max_ value would result in lower mixed venous O_2_ contents and higher systemic O_2_ extractions. Thus, when comparing the effect of different studies on variables determined by calculation from the $\dot{\text{V}}$O_2max_, it is crucial to have into consideration the averaging strategy applied in each investigation. Therefore, these facts may contribute to explain some of the variability in the literature when secondary-outcome variables have been based on $\dot{\text{V}}$O_2max_ values computed with large or small averaging strategies.

Based on the reproducibility of measurements, no particular sampling strategy seem superior for assessment of $\dot{\text{V}}$O_2max_. Notwithstanding, given that the $\dot{\text{V}}$O_2max_ can be sustained for a limited time, a shorter averaging strategy offers a higher probability for capturing the true $\dot{\text{V}}$O_2max_, while facilitating the identification of the plateauing criteria (Martin-Rincon et al., 2019). Thus, when using modern metabolic carts, averaging intervals including those between 15 and 20 breaths (or seconds) are preferable as a compromise between capturing a $\dot{\text{V}}$O_2_ value close to the true $\dot{\text{V}}$O_2max_ and identifying the plateau. So far, the time resolution of the assessment of cardiac output and blood gases has not been sufficient as to clarify what factors determine the plateau phenomenon.

## Author Contributions

Both authors contributed similarly and approved the final version of the manuscript.

## Conflict of Interest

The authors declare that the research was conducted in the absence of any commercial or financial relationships that could be construed as a potential conflict of interest.
